# Adaptation of cell spreading to varying fibronectin densities and topographies is facilitated by β1 integrins

**DOI:** 10.3389/fbioe.2022.964259

**Published:** 2022-08-10

**Authors:** Enrico Domenico Lemma, Zhongxiang Jiang, Franziska Klein, Tanja Landmann, Kai Weißenbruch, Sarah Bertels, Marc Hippler, Bernhard Wehrle-Haller, Martin Bastmeyer

**Affiliations:** ^1^ Zoological Institute, Karlsruhe Institute of Technology (KIT), Karlsruhe, Germany; ^2^ DFG-Center for Functional Nanostructures (CFN), Karlsruher Institut für Technologie, Karlsruhe, Germany; ^3^ Institute of Applied Physics, Karlsruhe Institute of Technology (KIT), Karlsruhe, Germany; ^4^ Department of Cell Physiology and Metabolism, University of Geneva, Geneva, Switzerland; ^5^ Institute of Biological and Chemical Systems—Biological Information Processing, Karlsruhe Institute of Technology, Karlsruhe, Germany

**Keywords:** cell spreading, cell signalling, fibronectin, integrin, micropatterning

## Abstract

Cells mechanical behaviour in physiological environments is mediated by interactions with the extracellular matrix (ECM). In particular, cells can adapt their shape according to the availability of ECM proteins, e.g., fibronectin (FN). Several *in vitro* experiments usually simulate the ECM by functionalizing the surfaces on which cells grow with FN. However, the mechanisms underlying cell spreading on non-uniformly FN-coated two-dimensional substrates are not clarified yet. In this work, we studied cell spreading on variously functionalized substrates: FN was either uniformly distributed or selectively patterned on flat surfaces, to show that A549, BRL, B16 and NIH 3T3 cell lines are able to sense the overall FN binding sites independently of their spatial arrangement. Instead, only the total amount of available FN influences cells spreading area, which positively correlates to the FN density. Immunocytochemical analysis showed that β1 integrin subunits are mainly responsible for this behaviour, as further confirmed by spreading experiments with β1-deficient cells. In the latter case, indeed, cells areas do not show a dependency on the amount of available FN on the substrates. Therefore, we envision for β1 a predominant role in cells for sensing the number of ECM ligands with respect to other focal adhesion proteins.

## Introduction

Mechanotransduction is a complex phenomenon through which cells convert mechanical stimuli from the extracellular environment to biochemical signaling events, ultimately adapting their shape and behaviour to changing environmental cues (Vogel and Sheetz, 2006). *In vivo*, cell adhesion, migration, and differentiation are predominantly influenced by the differential organisation of the extracellular matrix (ECM). A variety of factors are involved in shaping the mechano-signaling response, ranging from the architecture and stiffness of the ECM to the type and density of ECM proteins. Several studies have indicated how mechanical features of the cellular environment play a role in determining the dynamics of focal adhesions (FAs), which in turn activate regulatory pathways involved in cell mobility, proliferation and differentiation (Stutchbury et al., 2017; Matellan and del Rıó Hernández, 2019). FAs are multi-protein complexes consisting of ECM-ligands, transmembrane receptors and more than 160 different intracellular scaffold and signalling proteins (Geiger et al., 2009). They dynamically enter and leave the cell adhesion complex, depending on the function and maturation of the cell-matrix adhesion structure. Among different types of cell surface receptors that can interact with components of the extracellular matrix, the integrin family is one of the most important for the cell-ECM connection (Bachmann et al., 2019). Integrins are heterodimeric proteins consisting of the α and β subunits, each presenting variants which lead to more than twenty integrin types. Integrin-dependent cell-matrix adhesions are not just the physical connection to the ECM, but also transmit signals and forces from the extracellular environment into the cell by outside-in signalling (Matthews et al., 2006; Xia and Kanchanawong, 2017; Kechagia et al., 2019; Sun et al., 2019).

Cell adhesion and subsequent spreading are essential for the growth and function of adherent cells. The cell spreading process on ECM-coated surfaces requires a recognition and signalling component and depends on building the cell/ECM contacts and its dynamics, as well as on the subsequent reorganization of actin cytoskeleton and generation of actomyosin contractility (Murrell et al., 2015). The spreading process is a continuous event (Brill-Karniely et al., 2014) that consist of three phases (Döbereiner et al., 2004; Frame and Norman, 2008): 1) building of cell/ECM contact, which might be integrin-independent; 2) fast increase of the contact area involving assembly of focal complex and the FAK-dependent spreading; 3) stabilization and maybe polarization of the cell characterized by Rho-induced contraction and formation of stable adhesion.

How cells sense the physical properties of the environment (e.g., elasticity) is extensively reviewed (Geiger et al., 2009; Yang et al., 2017), but not much is yet known about how cells count and integrate the amount of the ECM molecules in their surroundings. Cell-spreading experiments on planar surfaces with varying physical parameters (e.g., rigid or elastic) and homogenously coated with different concentration of the studied protein show different relationships between cell size and the density of supplied ligands. This has led to the generally accepted conclusion that cell response to increased ECM-density is quite variable according to experimental conditions. Cell spreading can be a biphasic process characterized by reaching a maximum at an optimal ECM coating density and smaller cell size at lower ECM densities (Rajagopalan et al., 2004; Le Saux et al., 2011). Alternatively, it has also been reported that cell size just increases with increasing ligand density to reach a maximum constant value (Dubin-Thaler et al., 2004). These degree of spreading showed to be dependent also on substrates stiffness, which is another parameter able to influence cell area (Tee et al., 2011), potentially due to the consolidation phase of the spreading process. In this latter case, an optimal balance between substrate stiffness influencing the signalling response and ECM ligand density which determines the connectivity leads to maximal spreading (Engler et al., 2003).

However, not only the aforementioned variables but also the geometrical distribution of ECM ligands play a role in the cell behaviour. As the techniques to pattern ECM proteins on substrates improve (Li et al., 2019), it has been possible to show that the geometry of ligand distribution is essential for cell growth (Théry et al., 2006) and differentiation (Wang et al., 2019). Moreover, surface patterning represents a different route to vary ligand density on the substrate surface in alternative, or addition, to ECM protein dilution. In this case, the local density of the ligand remains maximal, but the average number of molecules per unit area may decrease according to the spacing of the functionalized patterns, both at the micro- (Lehnert et al., 2004) and at the nano-scale (Deeg et al., 2011; Zarka et al., 2019). In this case, a monotonically increasing cell size has been reported (Lehnert et al., 2004). However, a comprehensive overview of how cells arrange their area in response to 1) chemically diluted and 2) geometrically distributed ECM molecules in systematically defined experimental conditions is, to the best of our knowledge, still missing.

Here, we studied the capability of several cell lines to sense the amount of ECM ligands on flat and micropatterned substrates and to adjust their spreading accordingly. In comparison to other approaches (Cavalcanti-Adam et al., 2008; Satav et al., 2015), our investigation clarifies the impact of fibronectin (FN) density variation, due to both 1) dilution and 2) geometrical patterning. A direct correlation between FN availability and cell spreading area was observed. Depending on the cell line, spreading area values saturated after a defined amount of FN. Interestingly, the cell spreading was similar both when FN density was chemically controlled (i.e., *via* dilution on flat surfaces with denatured FN) and when it was geometrically regulated (i.e., *via* controlled patterning of FN micro-islands), and the monotonic increase was independent on the patterning technique. In all cases, the β1 integrin subunit showed to be responsible for the sensing of the FN amount, being distributed on all cell/substrate interfaces. This finding was confirmed by assessing the spreading of β1-deficient cells, whose area resulted to be independent on the FN amount. Furthermore, GD25-wt cells which were transfected to stably express the canonical β1A integrin isoform, namely GD25-β1A, again showed a correlation between FN and spreading area. Moreover, the distribution of several FA-related proteins was investigated to infer their role in sensing the ECM amount on flat and patterned substrates. Our results are also compatible with the mechanosensing-mechanosignalling model proposed by Stutchbury et al. (2017).

## Results

### Cell area monotonically correlates to different ligand density and substrate patterning

**FIGURE 1:**
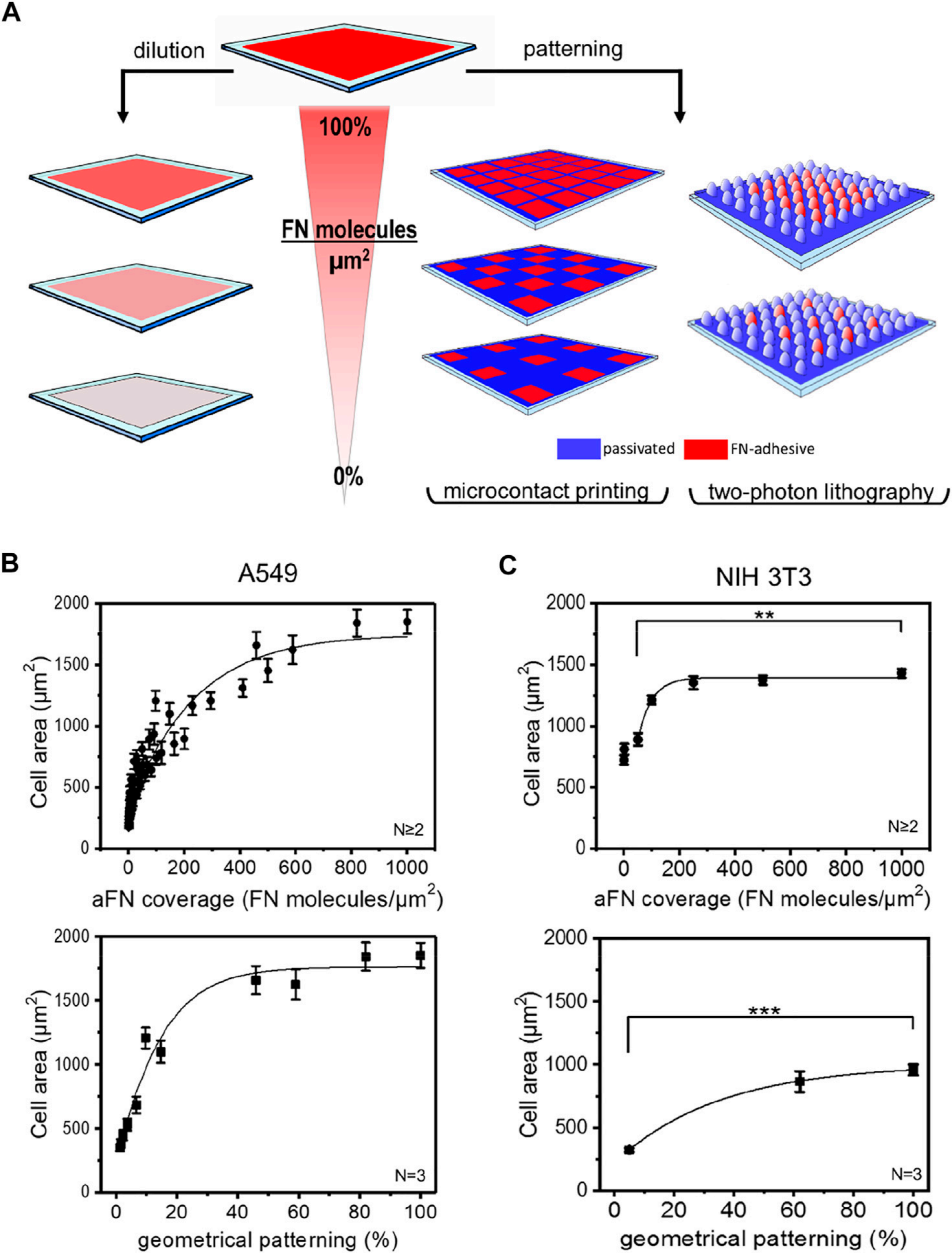
Microfabrication techniques and cell area spreading on differently FN-coated substrates. **(A)** Overview of the approach used to study the effects of ligand density on cell area spreading: flat surfaces were functionalized with different fibronectin (FN) dilutions, while micropatterns obtained *via* micro-contact printing (µCP) and two-photon lithography (2PL) were covered with undiluted FN. This strategy allowed for chemical and geometrical variation of FN density on substrates, respectively. **(B)** A549 cell areas measured on flat glass surfaces (top, circles) and on µCP patterns (bottom, squares). **(C)** NIH 3T3 cell areas measured on glass (top, circles) and on 2PL-patterned structures (bottom, squares).

In order to establish a possible correlation between FN coverage of the substrates and cells area, two strategies were followed (Figure 1A).

The first approach consisted in sequential dilutions of FN on flat glass substrates. These were obtained by mixing FN as received (aFN) at a concentration of 10 μg/ml with an appropriate amount of denatured FN (dFN) also at a concentration of 10 μg/ml. In this way, it was possible to maintain the total number of FN molecules adsorbed on the substrates surface at a constant value of approximately 1000 molecules/µm^2 23^; however, the eventual presence in the mixture of denatured FN molecules allowed for an effective reduction of the available binding sites for integrins (Supplementary Figure S1).

The second experimental condition to assess the spreading behaviour of cells in presence of controlled amounts of FN consisted in realizing microenvironments on which FN was not homogeneously distributed, but rather patterned at defined distances.

As the cellular mechanosensing may be studied by using several technologies (Ermis et al., 2018), we also addressed the question whether the micropatterning technique plays a significant role in cell spreading. We therefore used microcontact printing (µCP) and two-photon lithography (2PL) to realize geometrical patterns of aFN on 2D surfaces. Briefly, µCP is a soft-lithography technique which allows for serial replication of microstructures from a master stamp (Hu et al., 2018) of several differently inter-spaced islands of aFN. 2PL instead, is a direct laser writing technique which allows for rapid prototyping of two- and three-dimensional microstructures and patterns (Hippler et al., 2019; Lemma et al., 2019). In the case of 2PL, the patterned surfaces were realized following a two-step procedure: firstly, a patterned substrate was fabricated with a FN-repulsive material, and subsequently the areas to be functionalized were fabricated with FN-adhesive polymeric photosensitive material (PETA, see methods section for details). In particular, two pillar-based patterns were designed to allow a final FN-covered area of ≈60% and ≈5% of the overall surface (Supplementary Figures 2A,B).

Independently of the technique used for patterning, in order to avoid possible interference due to serum-adsorbed ECM molecules cells were seeded on the substrates in serum-free medium, after 1-h protein coating. After 3 h from seeding, cell spreading was complete and cell areas were plotted against the dilution- or pattern-induced FN availability. Figures 1B,C show the cell area vs. aFN availability for A549 human lung cancer cells and NIH 3T3 mouse fibroblasts on flat and patterned substrates *via* µCP and 2PL, respectively.

Interestingly, for flat substrates a clear monotonic trend is shown. In particular, cell area ranged between a minimum value of ≈500 µm^2^ for 5% aFN density and a maximum value of ≈1800 µm^2^ for A549 cells and of ≈1400 µm^2^ for NIH 3T3. The maximum value plateaus are reached at approximately 50% aFN dilution for A549 and at approximately 20% for NIH 3T3. This suggests that different cell lines show different saturation values for cell areas, as also shown by dilution experiments for buffalo rat liver (BRL) and mouse melanoma (B16) cells (Supplementary Figure 3A).

A similar monotonic increase in cell areas is also observed on micropatterned surfaces, where µCP aFN islands and 2PL-fabricated micro-pillar patterns only allowed for limited stretching of cells in a geometrical-patterning-dependent fashion (from ≈500 µm^2^ to ≈1750 µm^2^ for A549 cells, and from ≈300 μm^2^ to ≈900 µm^2^ for NIH 3T3, Figures 1B,C).

As a control for the suitability of the materials used in 2PL for cell area measurements, NIH 3T3 cells were also cultured on flat squares of 200 µm × 200 µm (width x length) with diluted FN (Supplementary Figure 3B), showing again a clear monotonic trend saturating at ≈20% of FN availability, similar to the one reported in Figure 1C. Smaller spreading areas measured on 2PL-fabricated squares might be due to the higher Young´s modulus of glass with respect to PETA (Lemma et al., 2017), despite both substrates provide stiffness values compatible with certain physiological environments such as bone surfaces (Akhmanova et al., 2015). However, the relative increase of cell area between the 5% aFN dilution and the 100% aFN coverage is similar in both cases (≈61% for bare glass, ≈65% for PETA substrates).

Given the similarity of results for the two patterning techniques (i.e., µCP and 2PL), in the following both were used independently for further investigation steps. In general, the versatility of µCP allows for modulating FN islands in a wider range with respect to 2PL, especially for low geometrical densities, while the realization of the master stamps requires longer procedures in comparison to 2PL.

### Patterned or diluted ligand density induce similar cell spreading

**FIGURE 2:**
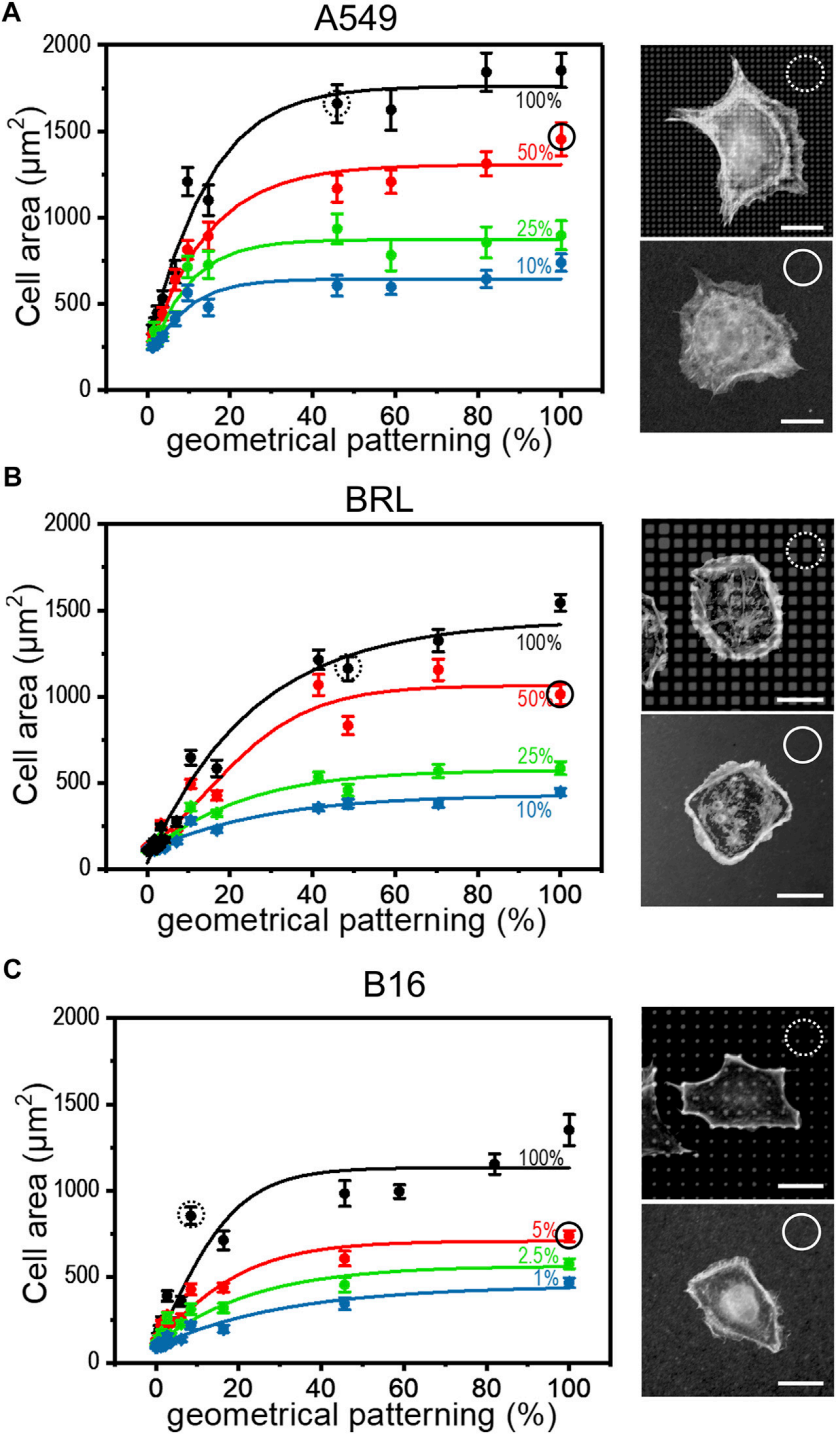
Cell spreading depends on FN availability. The aFN dilution and the geometrical pattern approaches were merged to show that the cell spreading areas have the same trend independently in both cases. Representative cells for 50% aFN dilution on flat surfaces (continuous circle) and undiluted aFN on 50% patterns (dotted circles) are shown on the right of each cell area graph. Average cell spreading area values indicated in dotted and continuous circles are statistically non-significant. **(A)**: A549 cells. **(B)**: BRL cells. **(C)**: B16 cells.

The strategies introduced in the previous paragraph for controlling surface aFN density, i.e., dilution with dFN and micro-patterning, were then combined to assess whether cells were able to recognize the total amount of available aFN. In particular, patterns made *via* µCP were functionalized not only with aFN, but also with several mixing ratios of aFN and dFN, respectively. Cell area measurements highlighted that the monotonic cell area increase takes also place in these functionalization conditions for the previously used epithelial cell line A549, and mesenchymal cell lines like BRL or B16 (Figures 2A–C). A statistical analysis showed that cell areas on patterns quantitatively resemble areas on flat substrates with a comparable amount of aFN (dotted and continuous circles in Figure 2A). Thus, if for a given cell line the spreading area reaches a plateau at 50% aFN on flat substrates (e.g., for A549 and BRL), then lower amounts of aFN will induce the same spreading either 1) by further dilution with dFN, or indifferently 2) by further patterning, given that the final density of aFN per unit area is the same. This behaviour was shown to be valid independently on the threshold at which the plateau is reached (e.g., B16 cells), and suggests that cell mechanosensing during spreading acts independently of how the underlying ECM protein is distributed, but only considers the total amount of protein which can be used by cells for FAs.

### β1 integrin is ubiquitous in focal adhesions on patterned substrates

The α5β1 integrin is also known as “fibronectin receptor” due to the specificity of the β1 subunit to bind the RGD sequence of FN. For this reason, it is hypothesized that β1 integrin has a central role in sensing the total amount of FN molecules on the substrates. Thus, the β1 subunit in NIH 3T3 cells in different conditions of aFN dilution and patterning was stained.

**FIGURE 3:**
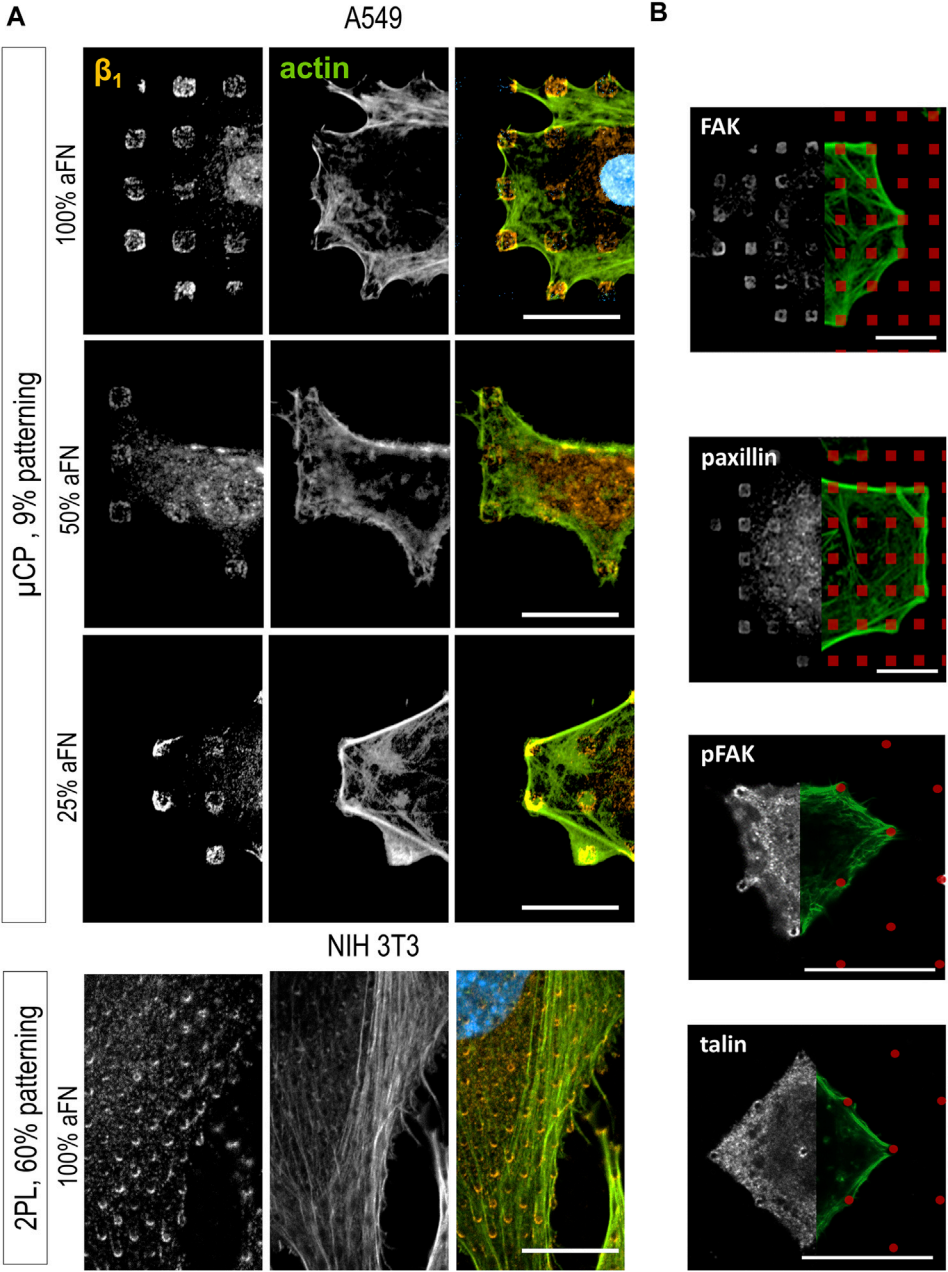
Cell spreading is β1-mediated. **(A)** β1 integrin is detected centrally and peripherally on patterns, independently on aFN dilution, fabrication system and cell line. **(B)** Other proteins involved on FAs are detected only peripherally (FAK, paxillin, pFAK), or centrally and peripherally (talin). FN-coated patterns are red-coloured for eye guidance. Scalebar 20 µm.

On flat substrates, the β1 fluorescence signal was diffusely distributed in the cells, with a certain clustering at peripheral FAs, independently of the aFN density (Supplementary Figure 4A). However, patterns reveal that β1 is indeed ubiquitously present within FAs. Indeed, Figure 3A shows that β1 is present on all the micro-islands and micro-pillars on which cells spread, where the local density of aFN is highest. Once again, the observation is independent 1) on the amount of aFN dilution, 2) on the cell line, and 3) on the patterning technique.

Further confirmation of this behaviour was obtained by analysing another set of patterns, namely 25 × 25 µm areas also obtained with 2PL. They were either 1) flat and functionalized with 100% aFN, or 2) patterned with micro-pillars to achieve a ≈60% aFN density per unit area when incubated with undiluted aFN (Supplementary Figures 4B,C). In this case, NIH 3T3 cells spanned over the whole available area and could not spread further, as the structures are surrounded by passivating TPETA. Thus, cell area was restricted to 625 µm^2^, yet β1 integrin was distributed both in the periphery and in the centre of cells (Supplementary Figures 4D,E). This observation is expected, and interestingly shows that β1 integrin ubiquitous location is not affected by the several single-cell and collective behaviours related to mechanosensing and mechanosignalling under physical constraints (Toyjanova et al., 2015; Ladoux and Mège, 2017; Mamidi et al., 2018).

The importance of β1 for sensing the amount of available aFN was further supported by the results of immunohistochemical staining of other proteins involved in the mechanotransduction pathway. Indeed, the mechanosensing machinery of cells comprises a number of proteins which mainly 1) provide the physical link between the FAs and the actin cytoskeleton, and 2) activate signalling pathways (e.g., the Rho/ROCK pathway) which lead to cytoskeleton rearrangements and ultimately to cell migration (Stutchbury et al., 2017). Integrins alone are not sufficient to induce a reaction to biomechanical cues in cells, but their role should be evaluated in the context of the other FAs proteins (Jansen et al., 2017). Thus, FAK, pFAK, paxillin, phosphorylated paxillin, kindlin2, α-actinin, talin and vinculin distribution in cells spread on substrates was studied. Most of them mainly showed a peripheral distribution on patterns (Figure 3B and Supplementary Figure 5). Only talin was ubiquitously distributed on cell/substrate contacts (Figure 3B), i.e., it was co-localized with β1, which is in agreement with models describing talin as a binding protein between actin fibres and integrins, together with vinculin (Humphries et al., 2007; Critchley and Gingras, 2008).

### GD25-wt cells do not show area vs. ligand density correlation, in contrast to GD25-β1A

**FIGURE 4:**
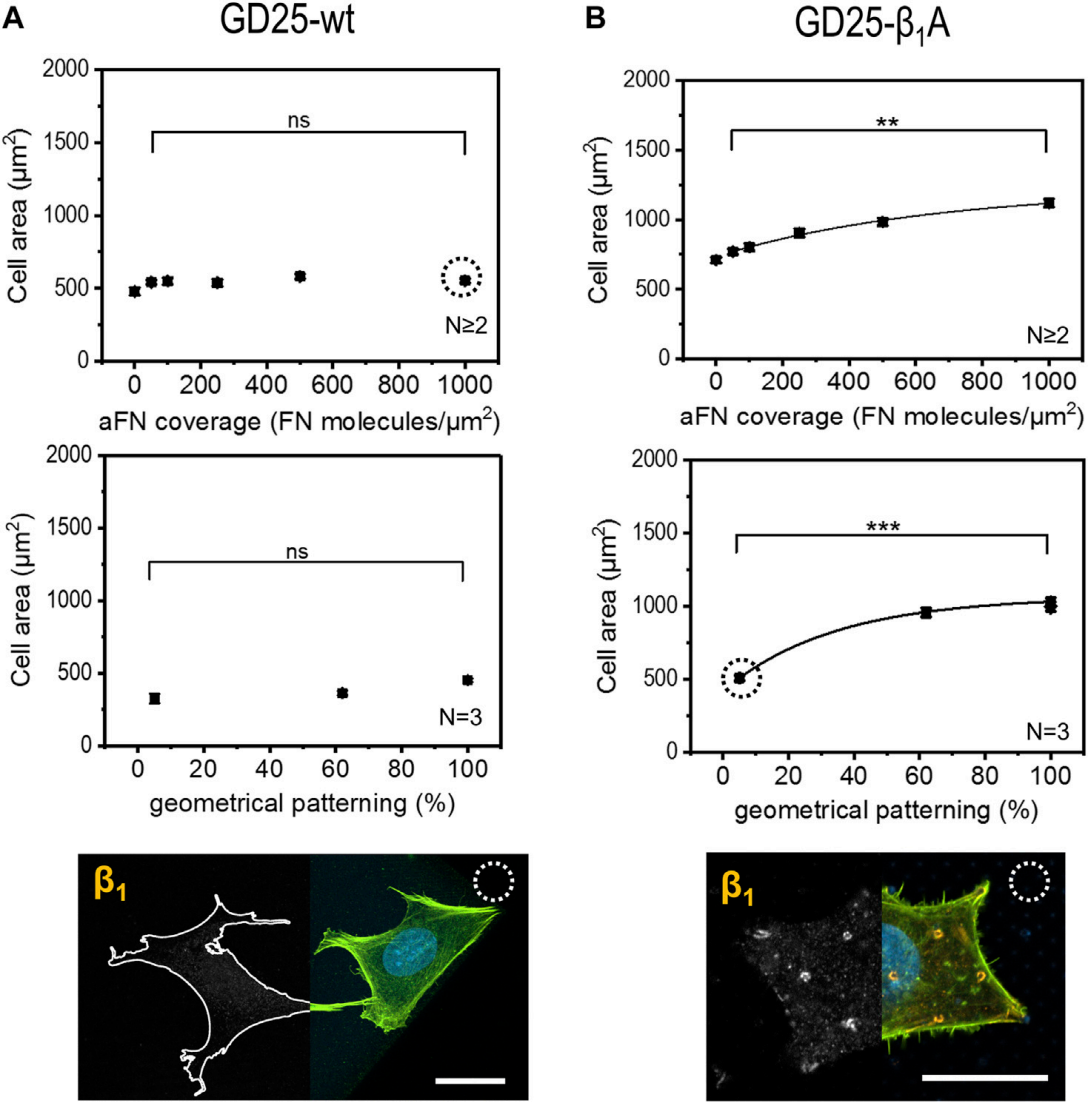
Cell area dependency on FN availability is absent in GD25-wt cells and is restored in GD25- β1A.**(A)** β1-deficient cells GD25-wt lack any increasing trend of spreading area, either on aFN diluted surfaces or patterns. Immunocytochemical stainings show the absence of β1 integrins (cell contours were drawn for eye guidance). **(B)** GD25-β1A cells show a restored spreading area trend, as well as β1 clusters on patterned surfaces centrally and peripherally. Scalebar 20 µm.

Given the evidence that cells use β1 integrin to build up FAs in correspondence of sites of aFN availability, a further step in the comprehension of FN sensing mechanisms was achieved by performing the aforementioned cell area measurements with a β1-deficient cell line, namely GD25-wt (Wennerberg et al., 1996). In order to account for the presumable reduced motility and spreading capability due to the absence of β1, cells were in this case cultured for 6 h and in DMEM with serum: cell area showed no increase with higher aFN availability, either in the case of dilution or geometrical patterning (Figure 4A, controls on 200 µm × 200 µm PETA squares at different aFN dilutions, and 3-h culture controls in DMEM without serum are shown in Supplementary Figures 6A,B). In the GD25-β1A clone (Wennerberg et al., 1996), the β1 subunit is stably re-expressed. Relatedly, the monotonical increasing trend in cell area was re-established (Figure 4B and Supplementary Figure 6C). In addition, β1 integrin showed a similar distribution as in the previously used cell lines (see also Supplementary Figure 6D). In order to directly compare experimental conditions between GD25 lines, also GD25-β1A cells were cultured for 6 h in DMEM with serum. Indeed, 3-h-lasting seeding shows not convincing results (Supplementary Figure 5E), thus strengthening the previous hypothesis that engineering β1 expression in GD25 cells induces these cells to form FAs and to spread over substrates slower than other immortalized cell lines. As a control, NIH 3T3 cells were also cultured in these modified experimental conditions, i.e., for 3 h and without FCS, showing no substantial variations in cell area with respect to 6-h culture with FCS (Supplementary Figure 6F).

**FIGURE 5:**
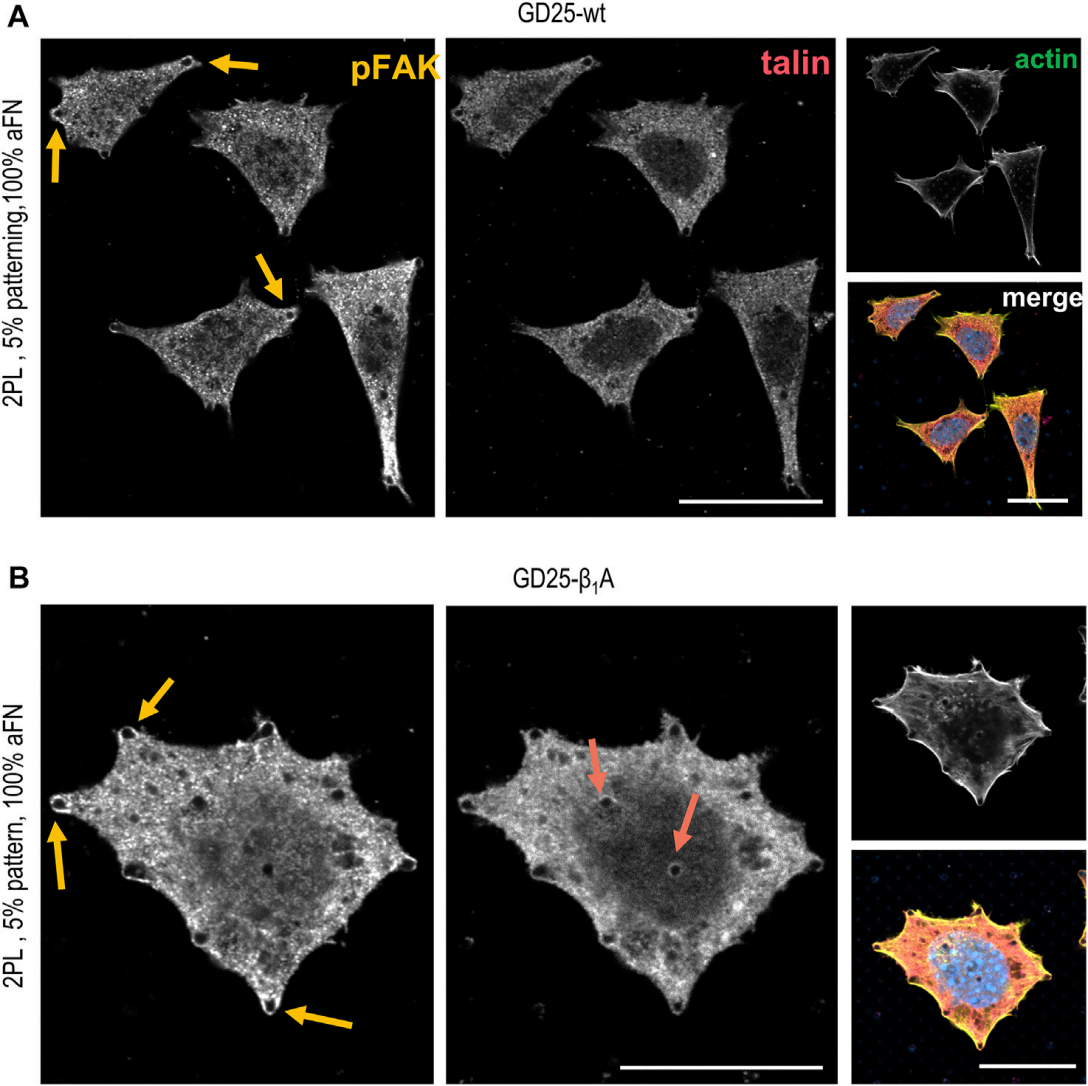
Phosphorylated FAK and talin stainings in GD25-wt and GD25- β1A.**(A)** GD25-wt cells on 2PL patterned substrates show only peripheral pFAK, while talin is not detectable. **(B)** Expression of talin in the central contacts of cells with the micro-pillars patterns is restored in the GD25-β1A line, which also shows peripheral pFAK. Scalebar 20 µm.

In order to assess whether the FAs proteins distribution was comparable to the previously used cell lines, the localization of talin and phosphorylated focal adhesion kinase (pFAK) was studied. Figures 5A,B depict talin and pFAK distribution in GD25-wt and GD25-β1A cells respectively, for the most extreme aFN densities, i.e., undiluted on flat substrates and on patterns with resulting 5% ligand density per unit area. A constant behaviour to be observed is the presence of talin in correspondence to FAs sites both peripherally and centrally, and a more confined presence of pFAK. This latter, instead, localizes predominantly in the outer regions of the cell body. Interestingly, patterned substrates clearly show that clustering of talin in GD25-wt is also impaired, while a certain activity of pFAK is still present at the peripheral micro-pillars. Even in this case, distribution is not altered in presence of physically constrained 25 × 25 µm flat or patterned substrates (Supplementary Figure S7 for GD25-β1A).

## Discussion and conclusion

Taken together, our results suggest that cells are able to sense FN density on substrates and to adjust their spreading area according to the number of available binding sites, independently whether these are homogeneously distributed or organized in geometrical patterns. Moreover, this behaviour is mainly driven by the β1 integrin subunit, with other FA proteins playing a secondary role. Indeed, understanding single-cell mechanics is a fundamental challenge in cell biology research because of the dynamic behaviour of every living organism, as well as for its countless practical applications in physiology and pathology (Jansen et al., 2015; Ruprecht et al., 2017; Matellan and Del Río Hernández, 2019). Despite advancements in the field that have allowed to disclose complex mechanisms underlying cell mechanical behaviour in a variety of conditions, it is still difficult to implement experimental setups to evaluate independently the several factors which play a role in cell mechanosensing. The complexity level of cells cannot be unlocked in single experiments, but it is increasingly easier to control parameters external to cells themselves, e.g., when dealing with ECM-like microenvironments. Improvements in microfabrication techniques have been allowing for more physiological and controlled conditions for cell culture and investigation of their interactions with the ECM. Our work intended to clarify the role of two conditions which are common to all complex systems for studying cell mechanosensing and are key to cell spreading: the effect of the amount of ECM protein which functionalizes the cell microenvironment and of the geometrical distribution of the protein itself in the microenvironment. In a relatively simple setup cells were subjected to spreading on different densities of FN on flat and micropatterned substrates. Results showed that independently on the substrate texture, cells were able to increase their area to a maximum threshold as a consequence of increase in protein availability. This behaviour suggests that cells may sense the amount of ligands and adjust their spreading to an optimal value for homeostasis, for that specific amount. This view implies an active role of cell mechanosensory machinery in 1) recognizing the FN availability and 2) provide a feedback to either induce further spreading or remain on a steady state condition. According to our evidence, β1 integrins seem to be responsible for integrating the amount of available FN together with talin: indeed, the localization of both proteins on areas at highest ligand availability confirm they are both needed to form the necessary bindings to the FN functionalized surfaces. Further evidence of this statement is the loss of cell area dependence on fibronectin density when β1 is impaired, e.g., in GD25-wt cells. The colocalization of talin clustering sites with β1 integrins and the counterpart peripheral localization of pFAK, despite not exhaustively clarifying the role of both proteins in mechanosensing, let us envision a direct involvement of the integrin-talin-actin axis in the quantification of ligand density on substrates. On the other hand, other FA proteins seem to be less directly involved in the recognition of availability sites of ligands. This let us envision for them an almost exclusive role in signalling and regulation downstream kinases and proteins of the Rho family (Schaller, 2010; Hytönen and Wehrle-Haller, 2016), and recruitment of the biochemical tools for cell spreading and locomotion, e.g., ultimately actin monomers to bundle in stress fibres. This hypothesis is in agreement with the model proposed by Stutchbury et al. (2017), according to which talin and FAK (together with paxillin and their phosphorylated version, and other minor FAs proteins like kindlin2 and α-actinin) belong to two distinct classes of FA proteins with different turnovers and roles in orchestrating cell spreading and migration.

## Materials and methods

### Fabrication *via* 2PL and µCP

2D and 3D structures microfabricated *via* 2PL were realized on 22 mm × 22 mm (length × width), 170 µm-thick glass coverslips. Substrates were cleansed with isopropanol, plasma-etched (air plasma, 10 min, TePla GmbH 100-E) in order to expose surface Si groups and silanized with a 1 mM solution of 3-(trimethoxysilyl) propyl methacrylate (CAS number 2530–85–0, from Sigma) in toluene. After 2 h incubation in silane solution, coverslips were rinsed twice in distilled water to complete the silanization reaction and dried with nitrogen.

A droplet (≈20 µl) of protein-repellent photoresist, i.e., trimethylolpropane ethoxylate triacrylate (TPETA, mean molecular weight 692Da, CAS number 28961–43–5, from Sigma) was cast on the silanized surface of the coverslip and exposed to a 780-nm femtosecond pulsed laser (Photonic Professional GT from Nanoscribe GmbH). After polymerization of the passivating structures, excess resist was removed in a bath of isopropanol. After drying, a droplet of FN-adhesive photoresist, i.e., pentaerythritol triacrylate (PETA, CAS number 3524–68–3, from Sigma) was cast on the sample, in order for the previously polymerized structures to be completely covered with the material. Writing of the designed structures was then performed and followed by excess resist development in a bath of 1:1 mixture of isopropanol:methyl-isobutyl-ketone. Structures were subsequently kept in isopropanol for sterilization and functionalized with FN.

The micro-islands were designed as cylinders with diameter 1 µm and height 500 nm, equally spaced by 3 µm center to center. The slicing ad hatching parameters were optimized for a ×63 oil immersion objective and were therefore set to 0.3 and 0.5 µm, respectively. The hatching was performed by using the “contour” option of the Describe software (from Nanoscribe GmbH) to improve shape fidelity. After fabrication and SEM inspection the pillars showed a more ellipsoidal shape with 1.5 × 0.7 × 0.7 µm dimensions (semiaxes a × b × c in Supplementary Figure S1B).

For both resists, the laser power ranged from 50 to 70% of the maximal available (≈55 mW) and the writing speed ranged from 2000 μm/s to 15000 μm/s.

Micropatterned substrates for µCP were prepared as described elsewhere (Lehnert et al., 2004). Briefly, a pattern consisted of two spot sizes (1 µm and 3 µm square) combined with eight distances (4, 5, 10, 15, 20, 25, 30 and 35 µm centre to centre), and was transferred to a coverslip coated with 20 nm gold. 1.5 mM solution of octadecylmercaptan (Sigma Aldrich) in ethanol was used as linker for binding of ECM protein to the surface. A hydrophilic alkanethiol (i.e., tri (ethylene glycol)-terminated alkanethiol, 1.5 mM in ethanol, Prochima) was used to block the remaining area and create an anti-adhesive background. The ECM-protein molecules selectively adsorbed onto the hydrophobic areas and formed a functionalized pattern.

### Functionalization and cell culture

Samples kept in isopropanol after 2PL were thoroughly washed with PBS (Ca^2+^- and Mg^2+^-free). For samples with non-reduced amount of active FN, 20 µl of a 10 μg/ml FN solution in PBS were placed on the area of the coverslip where structures had been fabricated. In the case of reduced active FN, denatured protein (heat treatment at 95°C for 30 min in PBS solution) was added to active FN to maintain always a final concentration of 10 μg/ml FN. As an example, for 500 molecules/µm^2^ of active FN, 10 µl of denatured FN (at 10 μg/μl) were added to 10 µl of active FN (at 10 μg/ml) and used to functionalize the structures.

In all cases, incubation was carried out for 1 h. All samples were then rinsed twice with PBS and cells were seeded.

Mouse Β16 melanoma cells, buffalo rat liver cells (BRL), human lung cancer cells (A549), and mouse embryo fibroblasts (NIH-3T3) were obtained from American Type Tissue Culture Collection (ATCC). GD25-wt and GD25-β1A cells were obtained from Prof. Bernhard Wehrle-Haller’s laboratory (Dept. of cell physiology and metabolism, University of Geneva, Switzerland). For routine culture, cells were grown in Dulbecco’s Modified Eagle Medium (DMEM), both supplemented with 10% FCS. GD25-β1A were cultured in DMEM+10%FCS and 10 mg/ml puromycin. Cells were always cultured in humidified atmosphere and 5% CO_2_. For detachment from culture flasks, cells were washed twice with PBS (Ca^2+^- and Mg^2+^-free) and removed from tissue culture plates by treatment with 0.1% trypsin/EDTA for 3–5 min. Dissociated cells were washed in DMEM with FCS for 3 min, and centrifuged (1000 rpm for 5 min). The cell pellet was resuspended and seeded [≈1 × 10 (Geiger et al., 2009) cells/cm^2^] on the patterned substrata in 2 ml DMEM without FCS (with the exception of GD25-wt and GD25-β1A) and cultured for three (Β16, BRL, A549, NIH-3T3) or six (GD25-wt and GD25-β1A) hours.

### Immunohistochemistry

Cells were fixed for 10 min with 4% PFA in PBS, washed three times with PBS containing 0.1% Triton X-100 (PBST) and incubated with primary antibodies for 1 h. The following primary antibodies were used: polyclonal anti-fibronectin (rabbit IgG, number F-3648 from Sigma) diluted 1:500 in PBS+1%BSA, monoclonal anti-pFAK Tyr397 (rabbit IgG, number 700255 from Invitrogen) diluted 1:300 in PBS+1%BSA, monoclonal anti-talin (mouse IgG, number TA1721 from Biotrend) diluted 1:100 in PBS+1%BSA, monoclonal anti-β1-integrin (rat IgG, number 550531 from BD Pharmingen) diluted 1:100 in PBS+1%BSA, monoclonal anti-vinculin (mouse IgG, number ab11194 from abcam) diluted 1:100 in PBS+1%BSA, polyclonal anti-kindlin2 (rabbit IgG, number K3269 from Sigma) diluted 1:250 in PBS+1%BSA, monoclonal anti-α-actinin (mouse IgG, number A7811 from Sigma) diluted 1:300 in PBS+1%BSA, monoclonal anti-phosphopaxillin (rabbit IgG, number ab32115 from abcam) diluted 1:500 in PBS+1%BSA. After incubation, samples were washed with PBST for three times and incubated with secondary antibodies for 1 h. The appropriate fluorescently coupled secondary antibodies (Cy3, Cy5, dilutions 1:400 and 1:200, respectively) were obtained from Dianova or Molecular Probes. Actin filaments were stained with AlexaFluor488-coupled phalloidin (diluted 1:200), nuclei were stained with DAPI (diluted 1:1000). After a final rinse, samples were mounted in Mowiol containing 1% N-propyl-gallate and analysed *via* laser scanning microscopy.

### Microscopy and cell area measurement

Confocal microscopy (LSM 800, LSM 510 and Apotome, Zeiss) was used for image acquisition. Pictures for cell area measurements were taken with ×40 magnification, oil immersion (N.A. 1.2), while FA proteins imaging required ×63 magnification (oil immersion) with N.A. 1.4 to maximize achievable resolution. Laser intensity and gain were adjusted according to the needed requirements. DAPI and Cy3 fluorescence were acquired simultaneously, in sequence to Cy5, and AlexaFluor488 signals. Digital zoom and image size were always adjusted to ensure fulfilment of the Nyquist criterion, colour depth was set to 8 bits.

Pictures were then opened in the open source Fiji-ImageJ software and AlexaFluor488 signal was shown in grey tones. A suitable threshold was then chosen in order to exclude background (usually pixels of greyscale values below 10), and to ensure that cell shape was not artificially diminished. Cell contours were then traced *via* the “wand tool” and cell surface (i.e., the pixel area comprised within the traced contours) was then automatically measured from the software.

In all manuscript figures, contrast and brightness were adjusted of the same amount exclusively for editorial purposes.

For scanning electron microscopy of patterns (Supplementary Figure 2A), a Zeiss Supra microscope was used and samples without cells on them were gold-sputtered (≈7 nm) prior to visualization.

### Statistical methods

All experiments were performed at least in duplicate, statistical significance was assessed between experiments performed in triplicate. In particular, average values of cell areas were compared, and statistical significance was proved with Student´s t-test, with the following *p*-values: ***p ≤ 0.001, **0.001 < *p*≤ 0.01, *0.01 < *p*≤ 0.05. Total number of analyzed cells was n ≥ 50 per each experimental condition.

## Data Availability

The datasets presented in this study can be found in online repositories. The names of the repository/repositories and accession number(s) can be found below: https://radar.kit.edu/.
