# Supplementary material for: Adaptation of cell spreading to varying fibronectin densities and topographies is facilitated by β1 integrins
Source: Front Bioeng Biotechnol. 2022 Aug 10;10:964259. doi: 10.3389/fbioe.2022.964259 (PMC9399860; doi:10.3389/fbioe.2022.964259)
Supplement: Supplementary file 1 [file Table1.DOCX]

**SUPPLEMENTARY INFORMATION**

**Adaptation of cell spreading to varying fibronectin densities and topographies is facilitated by β1 integrins.**

Enrico Domenico Lemma^1,*,‡^, Zhongxiang Jiang^1^, Franziska Klein^1,2^, Tanja Landmann^1^, Kai Weißenbruch^1^, Sarah Bertels^1^, Marc Hippler^1,3^, Bernard Wehrle-Haller^4^, Martin Bastmeyer^1, 5,*^

^1^ Zoological Institute, Karlsruhe Institute of Technology (KIT), Fritz-Haber-Weg, 4 – 76131 Karlsruhe, Germany
^2^ DFG-Center for Functional Nanostructures (CFN), Karlsruher Institut für Technologie, Wolfgang-Gaede-Str., 1 – 76128 Karlsruhe, Germany
^3^ Institute of Applied Physics, Karlsruhe Institute of Technology (KIT) Wolfgang-Gaede-Str., 1 – 76128 Karlsruhe, Germany
^4^ University of Geneva, Department of Cell Physiology and Metabolism, Rue Michel-Servet, 1 – 1211 Genève, Switzerland
^5^ Institute of Biological and Chemical Systems – Biological information processing, Karlsruhe Institute of Technology, Hermann-von-Helmholtz-Platz, 1 – 76344 Eggenstein-Leopoldshafen, Germany

^‡^Current address: Department of Engineering, Università Campus Biomedico di Roma, via Alvaro del Portillo 21 – 00128 Rome, Italy

*Corresponding authors: e.lemma@unicampus.it; martin.bastmeyer@kit.edu


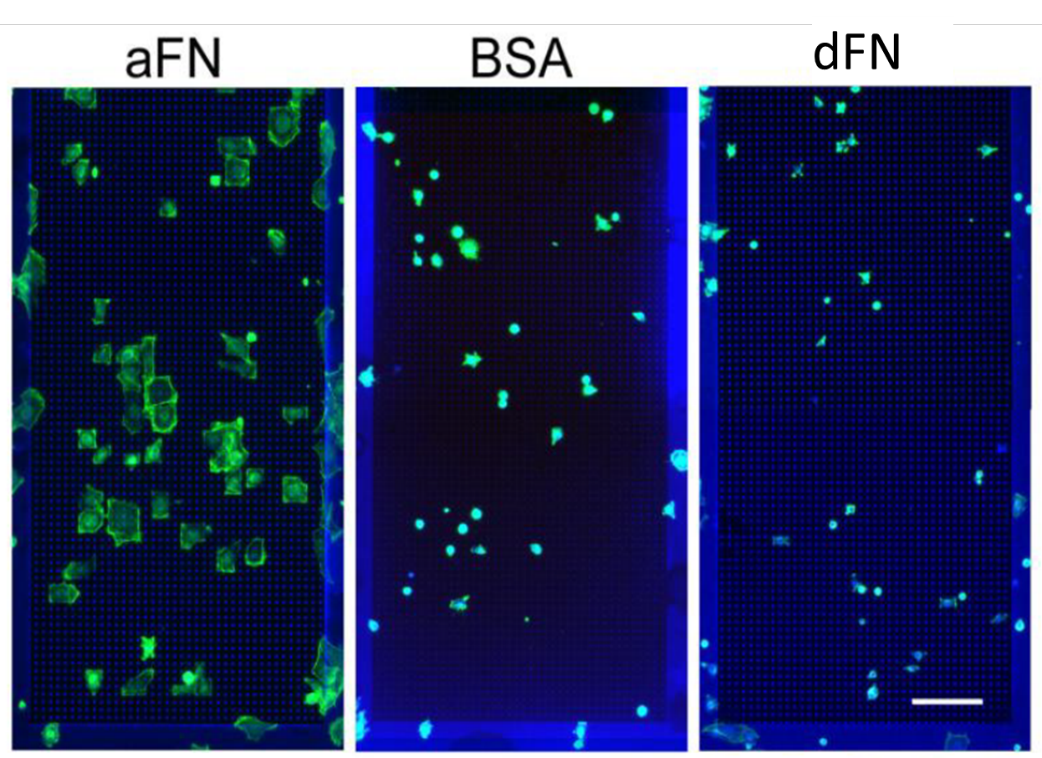


**Supplementary Figure 1.**

On µCP substrates, dFN shows the same effect of cells caused by bovine serum albumin (BSA), which does not show binding sites for cell spreading. Cells can still adhere to the functionalized substrate, but cannot develop mature focal adhesions and thus increase their areas, as it happens in the case of aFN.


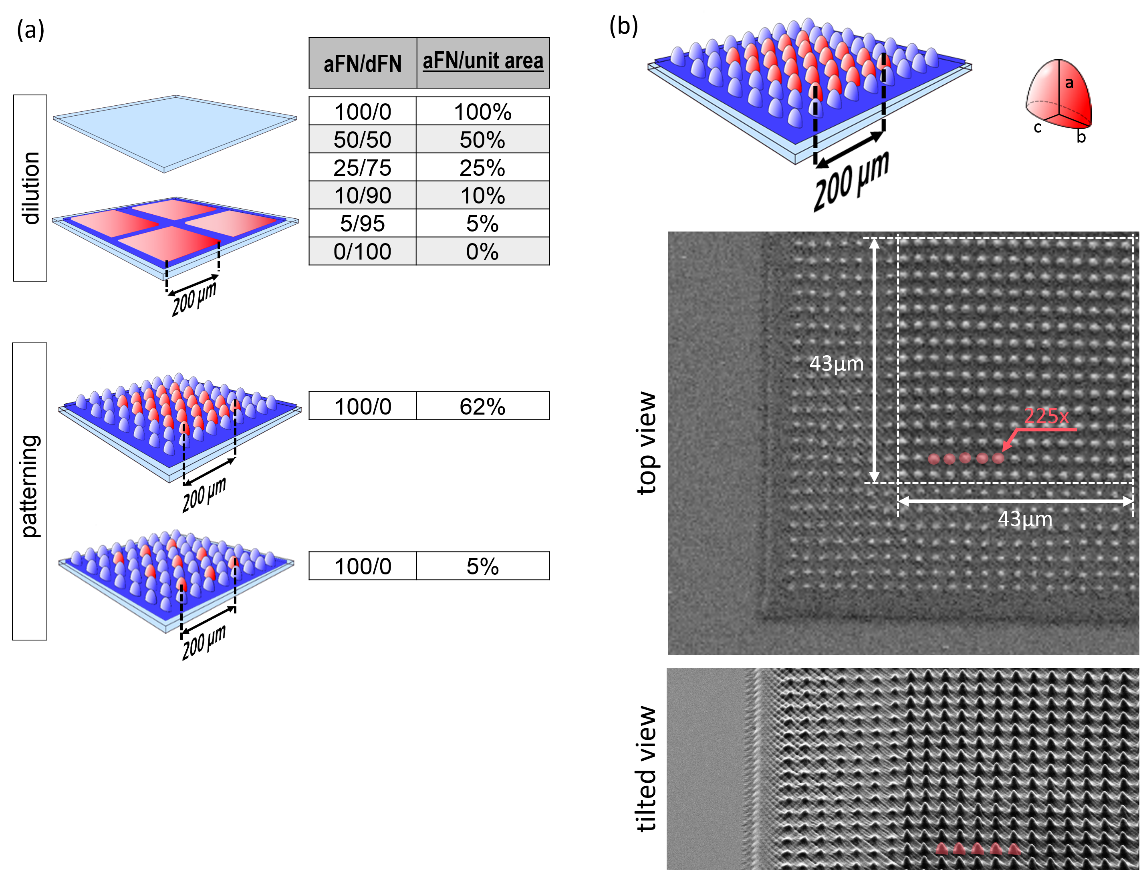


**Supplementary Figure 2.**
(a) Overview and schematic representation of the substrates and of the patterns used in the work.
(b) Detailed scheme (top) and scanning electron microscopy (bottom) of the 2PL-made patterns.


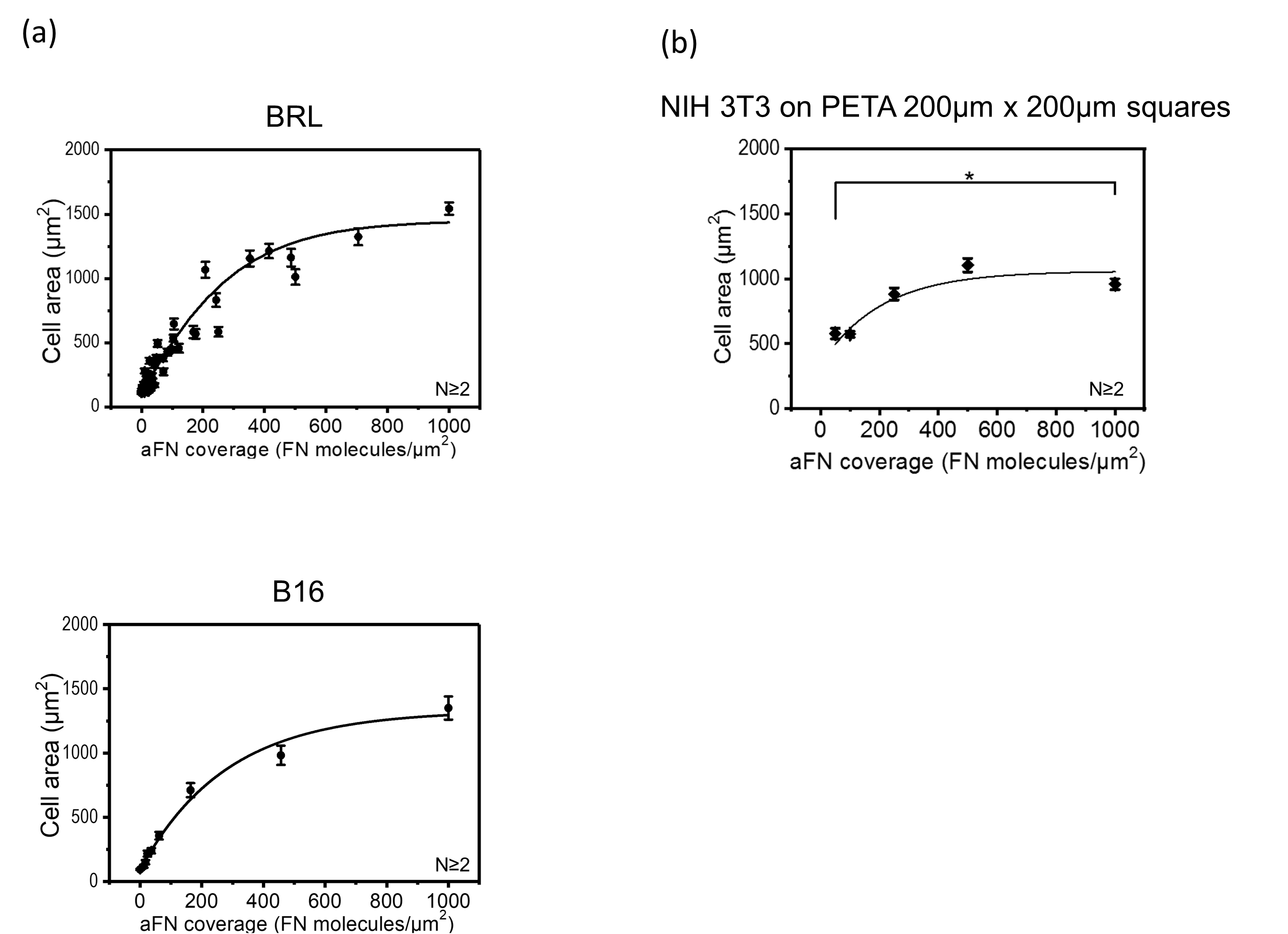


**Supplementary Figure 3.**
(a) BRL and B16 cells show an increasing trend in cell spreading area with increasing aFN concentrations.
(b) NIH 3T3 cells show a similar trend even when cultured on 2PL flat surfaces other than glass.


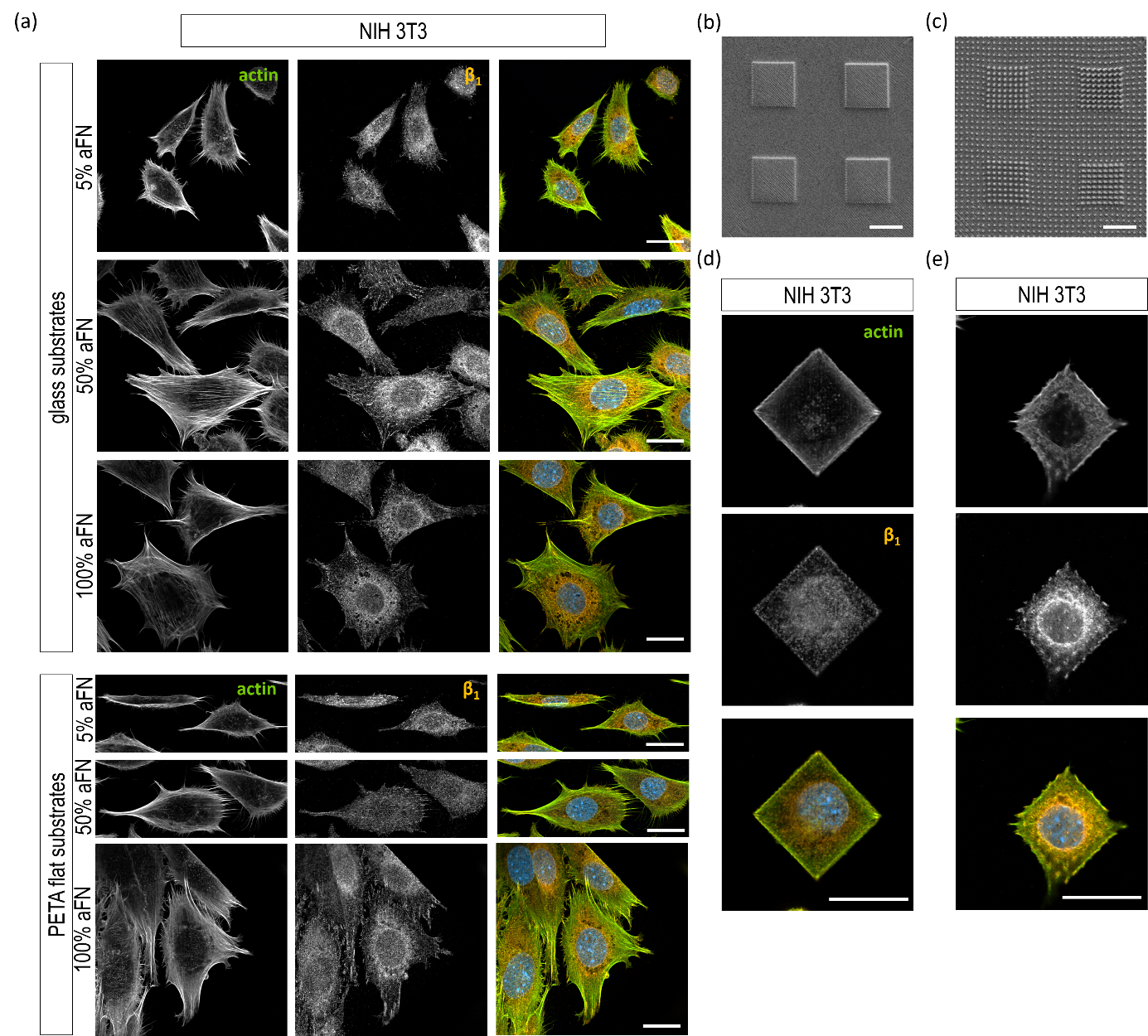


**Supplementary Figure 4.**
(a) Expression of β1 integrin on flat substrates (glass or 2PL-fabricated, 200µm x 200µm) in NIH 3T3 cells.
(b) and (c): scanning electron microscopy of 2PL-fabricated 25µm x 25µm squares, flat and patterned respectively.
(d) and (e): Expression of β1 integrin on 25µm x 25µm squares in NIH 3T3 cells.
Scalebar 20µm.


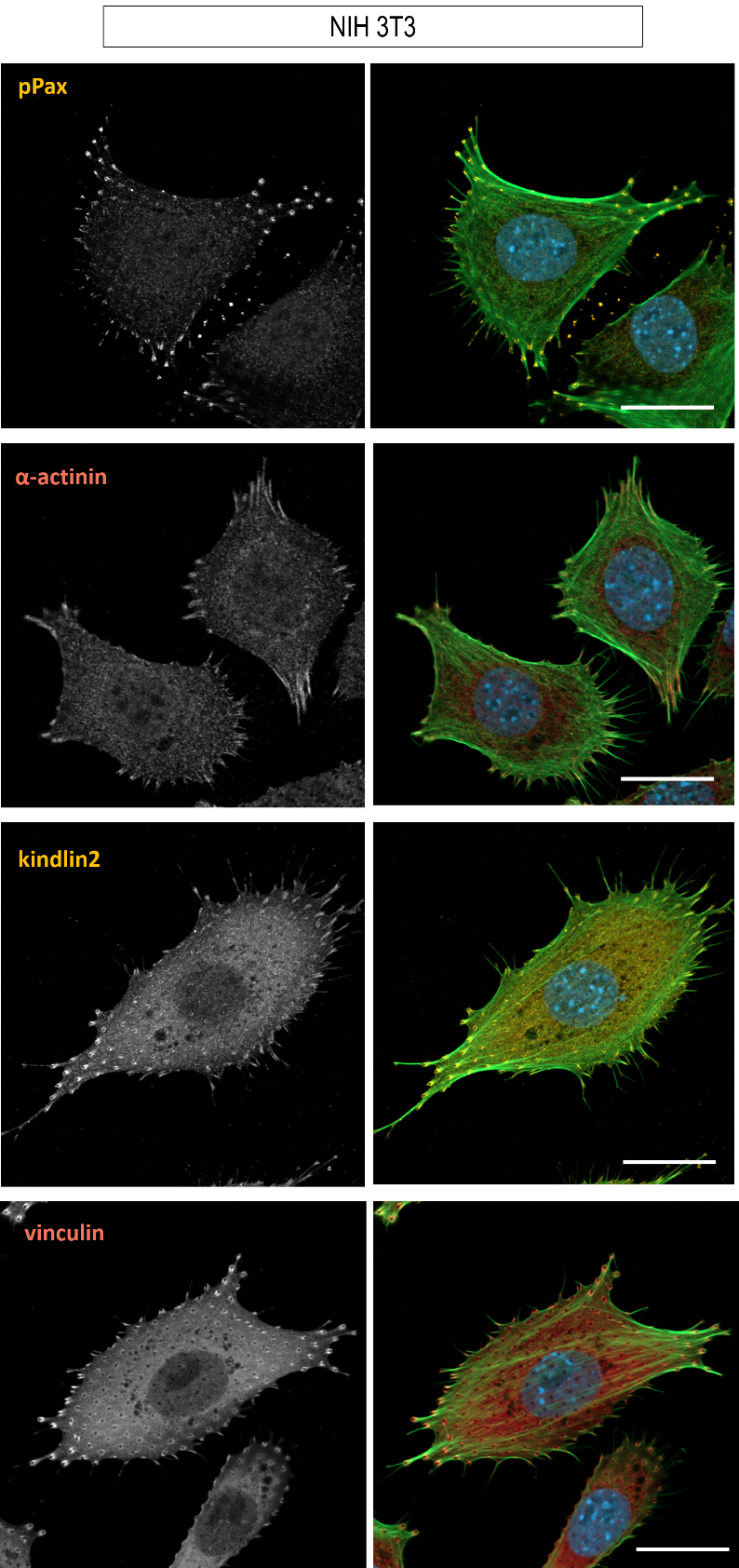


**Supplementary Figure 5.**Immunocytochemical staining of proteins involved in FAs in NIH 3T3 cells (phosphopaxillin, α-actinin, kindlin2 and vinculin from top to bottom) spread over 2PL patterns with ≈60% aFN.

**
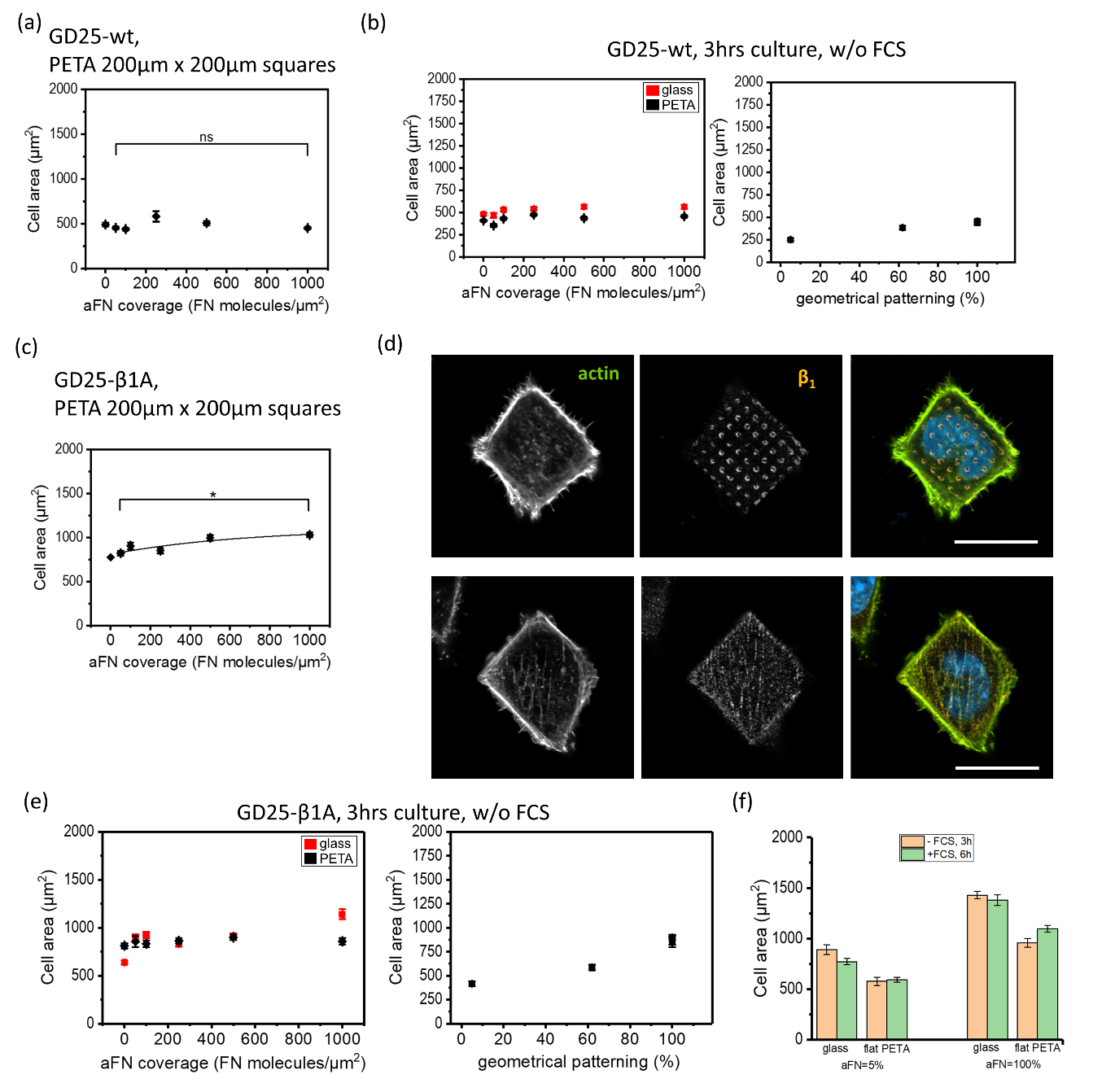
**

**Supplementary Figure 6.**(a) GD25-wt shows no spreading trend even on 200µm x 200µm PETA surfaces in presence of different aFN dilutions.
(b) Cell area of GD25-wt remains constant independently of aFN dilutions and geometrical patterns also after culturing for 3 hours.
(c) GD25-β1A cells show increasing areas with increasing aFN even on glass surfaces.
(d) β1 integrin is ubiquitous in GD25-β1A cells even on 25µm x 25µm squares.
(e) Three hours of culturing are not sufficient for GD25-β1A cells to properly spread on flat surfaces or patterns.
(f) Culture times do not significantly affect NIH 3T3 cell area for a given substrate condition (flat or patterned).


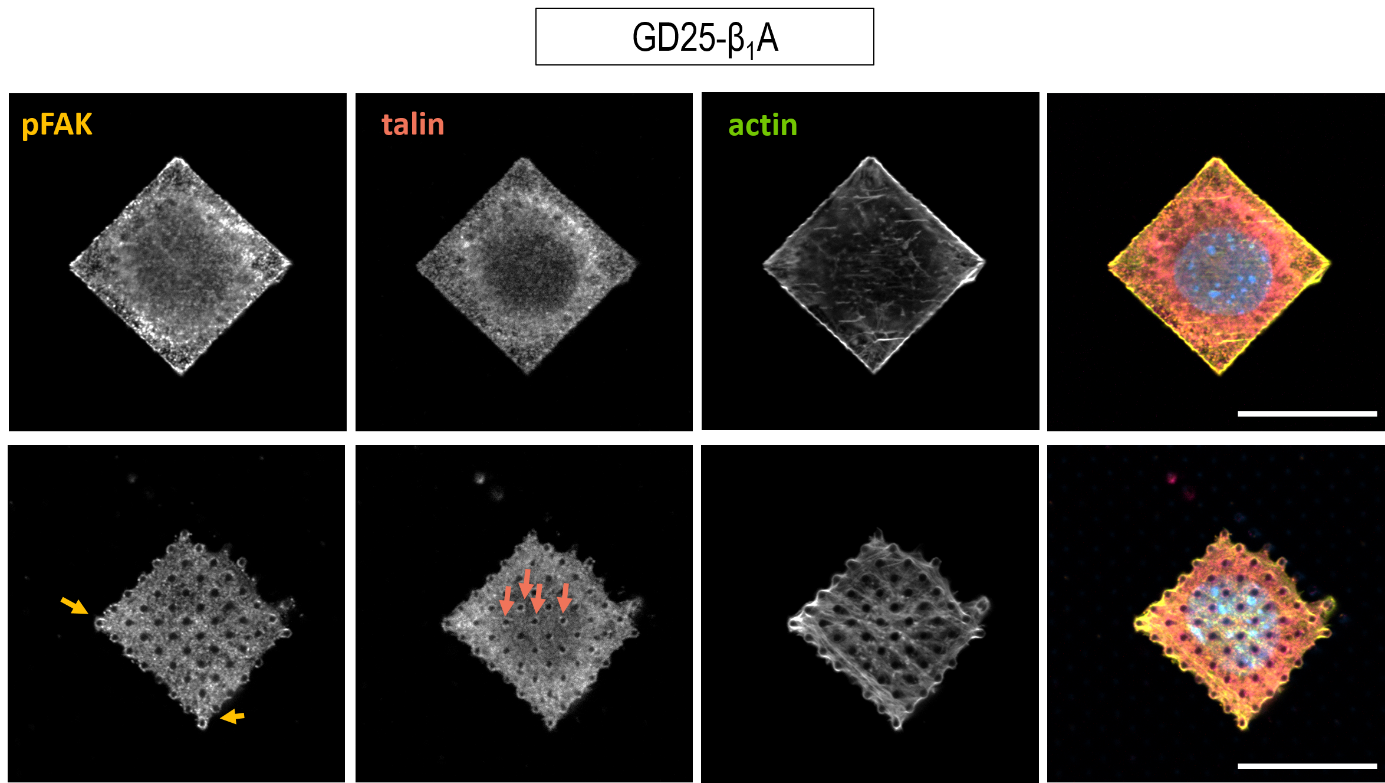


**Supplementary Figure 7.**Expression of pFAK (peripheral) and talin (peripheral and central) on 25µm x 25µm squares.
